# Zolpidem Use Associated With Increased Risk of Pyogenic Liver Abscess: A Case-Control Study in Taiwan

**DOI:** 10.1097/MD.0000000000001302

**Published:** 2015-08-14

**Authors:** Kuan-Fu Liao, Cheng-Li Lin, Shih-Wei Lai, Wen-Chi Chen

**Affiliations:** From the College of Medicine (K-FL), Tzu Chi University, Hualien; Department of Internal Medicine (K-FL), Taichung Tzu Chi General Hospital, Taichung; Graduate Institute of Integrated Medicine (K-FL, W-CC), China Medical University, Taichung; College of Medicine (C-LL, S-WL), China Medical University, Taichung; Management Office for Health Data (C-LL), China Medical University Hospital, Taichung; Department of Family Medicine (S-WL), China Medical University Hospital, Taichung; and Department of Urology (W-CC), China Medical University Hospital, Taichung, Taiwan.

## Abstract

The purpose of this study was to explore the association between zolpidem use and pyogenic liver abscess in Taiwan.

This was a population-based case-control study using the database of the Taiwan National Health Insurance Program since 2000 to 2011. We identified 1325 patients aged 20 to 84 years with the first-attack of pyogenic liver abscess as the cases, and 5082 patients without pyogenic liver abscess matched with sex, age, comorbidities, and index year of hospitalization for pyogenic liver abscess as the controls. Patients whose last remaining 1 tablet for zolpidem was noted ≤7 days before the date of admission for pyogenic liver abscess were defined as current use of zolpidem. Patients whose last remaining 1 tablet for zolpidem was noted >7 days before the date of admission for pyogenic liver abscess were defined as late use of zolpidem. Patients who never received 1 prescription for zolpidem were defined as never use of zolpidem. A multivariable unconditional logistic regression model was used to measure the odds ratio (OR) and 95% confidence interval (CI) to explore the association between zolpidem use and pyogenic liver abscess.

After adjustment for possible confounding variables, the adjusted OR of pyogenic liver abscess was 3.89 for patients with current use of zolpidem (95% CI 2.89, 5.23), when compared with those with never use of zolpidem. The adjusted OR decreased to 0.85 for those with late use of zolpidem (95% CI 0.70, 1.03), but without statistical significance.

Current use of zolpidem is associated with the increased risk of pyogenic liver abscess. Physicians should take the risk of pyogenic liver abscess into account when prescribing zolpidem.

## INTRODUCTION

Zolpidem is a nonbenzodiazepine hypnotic agent, which is mainly indicated for the treatment of insomnia. Initially, zolpidem was widely prescribed by physicians because of its favorable pharmacological properties according to the manufacturer's prescribing information, including rapid onset of action, reduction of sleep latency, increase of total sleep duration, reduction of nighttime awakening, few next-day effects on daytime well-being and morning coordination, and almost devoid of abuse and dependence potential.^1–5^ However, the growing evidence discloses that zolpidem may cause delirium, nightmares, hallucinations, and a significant potential for abuse and dependence.^6,7^ Some observational studies have disclosed that zolpidem may be associated with comorbidities, including motor vehicle accidents, stroke, hip fracture, glaucoma, and acute pancreatitis.^8–12^ In addition, 2 observational studies have disclosed that zolpidem may be associated with some infections such as rhinitis, pharyngitis, bronchitis, and pneumonia.^13,14^ In spite of no direct evidence showing the mechanism of zolpidem effect on infections, hypnotic-related immune suppression,^15,16^ hypnotic-related impairment of clearance of oral secretion during sleep,^17^ and hypnotic-related gastroesophageal reflux during sleep and further resulting aspiration^18,19^ can partially explain the mechanism of some infections such as upper airway infections. However, the association between zolpidem use and pyogenic liver abscess has never been studied.

Pyogenic liver abscess is an infective condition of the liver, which is mainly caused by a wide variety of infective agents. Its clinical course might range from totally cured to severe and life-threatening. The mortality rate of pyogenic liver abscess can be up to 8.2% in Chen et al's^20^ observational study using the database of the Taiwan National Health Insurance Program. To date, a number of well-documented risk factors including biliary tract disease, cirrhosis, diabetes mellitus, and renal disease are found to be associated with pyogenic liver abscess,^21–23^ but zolpidem-related pyogenic liver abscess has not yet been studied.

Based on the aforementioned review, we make a plausible hypothesis that there could be a link between zolpidem use and pyogenic liver abscess because of zolpidem-related infections. If the link really exists, physicians should consider the risk of pyogenic liver abscess among zolpidem users. Given that zolpidem is the most widely prescribed nonbenzodiazepine hypnotic agent in Taiwan,^24,25^ we conducted a case-control study using the database of the Taiwan National Health Insurance Program to explore the association between zolpidem use and pyogenic liver abscess.

## METHODS

### Design and Study Population

This was a population-based case-control study using the database of the Taiwan National Health Insurance Program. This program began in March 1995, which covered 99% of the total 23 million people living in Taiwan.^26^ The diagnosis of disease is based on the International Classification of Diseases, Ninth Revision, Clinical Modification (ICD-9 codes). The database is available for public access.

The number of patient identification has been scrambled to ensure confidentiality. The database includes personal socio-demographic status, such as sex and date of birth, and information on ambulatory care, inpatient care, emergency care, dental care, and prescription drugs. The details of the program were well written in previous high-quality articles.^27–30^ This study was approved by the Ethics Review Board of China Medical University and Hospital in Taiwan (CMU-REC-101-012).

### Participants

We used the inpatient claim dataset to identify patients aged 20 to 84 years with the first-attack of pyogenic liver abscess (ICD-9 code 572.0) during the period of 2000 to 2011 as the case group. The date of discharge with primary diagnosis of pyogenic liver abscess was defined as the index date. For each case of pyogenic liver abscess, 4 control patients aged 20 to 84 years without pyogenic liver abscess were randomly selected from the same database as the control group. Both case and control groups were matched with sex, age (every 5-year span), comorbidities, and index year of hospitalization for pyogenic liver abscess. Patients with prior diagnosis of amebic liver abscess (ICD-9 code 006.3) or liver transplantation (ICD-9 codes 996.82 and V42.7) before the index date were excluded from this study.

### Other Medications and Comorbidities

History of prescriptions for benzodiazepines available in Taiwan was included in this study. To decrease biased results, patients who had prescriptions of other nonbenzodiazepine hypnotic agents including zopiclone, zaleplon, and eszopiclone were excluded from this study. Comorbidities potentially related to pyogenic liver abscess before the index date were included as follows: alcohol-related diseases, biliary stone, chronic kidney diseases, diabetes mellitus, as well as chronic liver diseases, including cirrhosis, Hepatitis B, Hepatitis C, and other chronic hepatitis. All comorbidities were diagnosed with ICD-9 codes.

### Definition of Zolpidem Exposure

Because zolpidem has a short elimination half-life (approximately 2.1–2.4 hours) with no active metabolite, it does not cause an accumulating effect during repeated use.^3,31^ Therefore, we used 7 days as a cutoff point. Based on the prescription history, we can estimate the last remaining 1 tablet for zolpidem. Patients whose last remaining 1 tablet for zolpidem was noted ≤7 days before the date of admission for pyogenic liver abscess or those still having zolpidem tablets at the date of admission for pyogenic liver abscess were defined as current use of zolpidem. Patients whose last remaining 1 tablet for zolpidem was noted >7 days before the date of admission for pyogenic liver abscess were defined as late use of zolpidem. Patients who never received 1 prescription for zolpidem were defined as never use of zolpidem.

### Statistical Analysis

The distributions of sex, age, zolpidem use, benzodiazepines use, and comorbidities were compared between the case group and the control group using the χ^2^ test for categorized variables and *t* test for continuous variables. Variables found significantly related to pyogenic liver abscess in the univariable unconditional logistic regression model were further included in the multivariable unconditional logistic regression model. The odds ratio (OR) and 95% confidence interval (CI) were measured to explore the risk of pyogenic liver abscess associated with zolpidem use, benzodiazepines use, and comorbidities. We also explore the association between average daily dose of current use of zolpidem and the risk of pyogenic liver abscess. The average daily dose of zolpidem was calculated by using the total prescribed dose divided by total number of days supplied. Two strengths of zolpidem are available in Taiwan for oral administration as 6.25 mg (extended-release tablet) and 10 mg. Therefore, we used 10 mg as a cutoff point. We classified patients with current use of zolpidem into 2 subgroups: high dose group with average daily dose >10 mg and low dose group with average daily dose ≤10 mg. All data processing and statistical analyses were performed with the SAS software version 9.2 (SAS Institute, Inc, Cary, NC). A 2-tailed *P* value of <0.05 was considered statistically significant.

## RESULTS

### Characteristics of the Study Population

Table 1 shows the distributions of sex, age, zolpidem use, benzodiazepines use, and comorbidities between the case group and the control group. This study consisted of 1325 cases with pyogenic liver abscess and 5082 controls without pyogenic liver abscess, with similar distributions of sex and age. The mean age (standard deviation) of the study patients was 59.5 ± 14.5 years for the case group and 59.3 ± 14.5 years for the control group (*t* test, *P* = 0.60). The case group had higher proportions of current use of zolpidem (7.4% vs 1.8%), ever use of benzodiazepines (82.0% vs 75.2%), alcohol-related diseases (7.09% vs 5.45%), biliary stone (19.0% vs 13.0%), chronic liver diseases (33.5% vs 29.8%), and diabetes mellitus (45.4% vs. 39.8%) than the control group, with statistical significance.

**TABLE 1:**
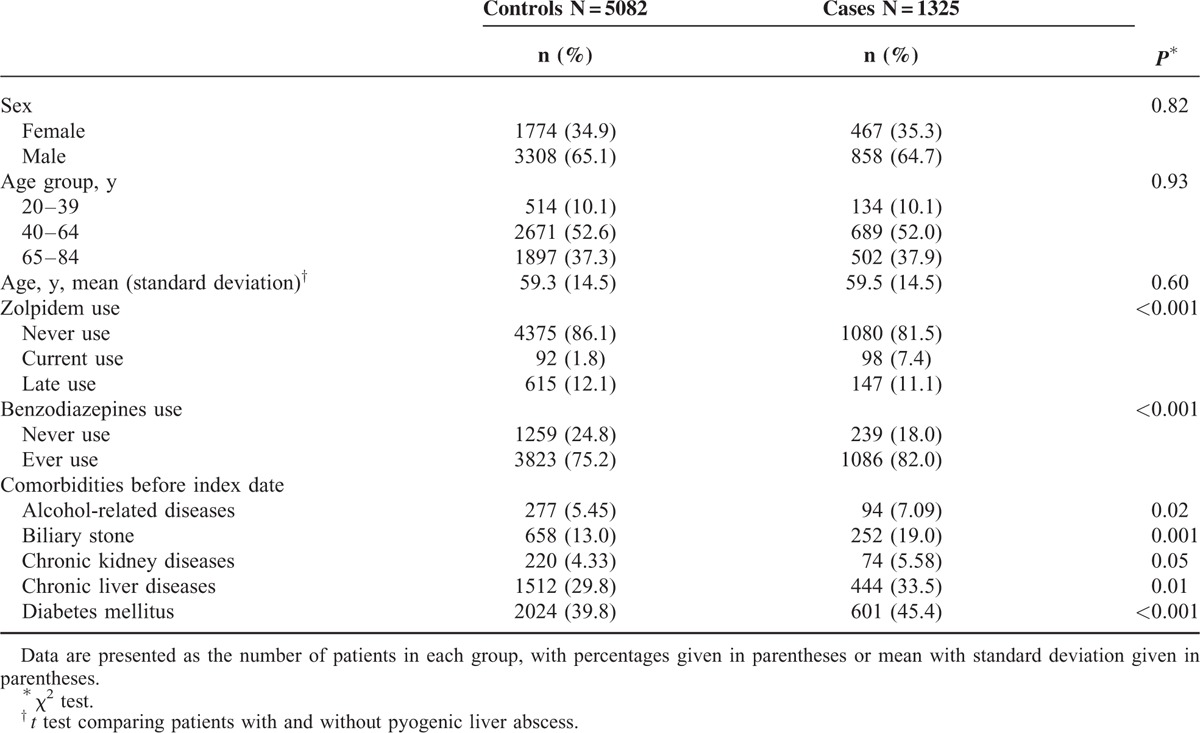
Characteristics Between Pyogenic Liver Abscess Cases and Controls

### Risk of Pyogenic Liver Abscess Associated With Zolpidem Use and Other Factors

Table 2 shows the risk of pyogenic liver abscess associated with zolpidem use, benzodiazepines use, and other comorbidities. After adjustment for possible confounding factors, the multivariable unconditional logistic regression model disclosed that the adjusted OR of pyogenic liver abscess was 3.89 for patients with current use of zolpidem (95% CI 2.89, 5.23), when compared with those with never use of zolpidem. The adjusted OR decreased to 0.85 for those with late use of zolpidem (95% CI 0.70, 1.03), but without statistical significance. In addition, benzodiazepines use (adjusted OR 1.32, 95% CI 1.13, 1.55), biliary stone (adjusted OR 1.57, 95% CI 1.33, 1.85), and diabetes mellitus (adjusted OR 1.21, 95% CI 1.06, 1.37) were other factors significantly associated with pyogenic liver abscess.

**TABLE 2:**
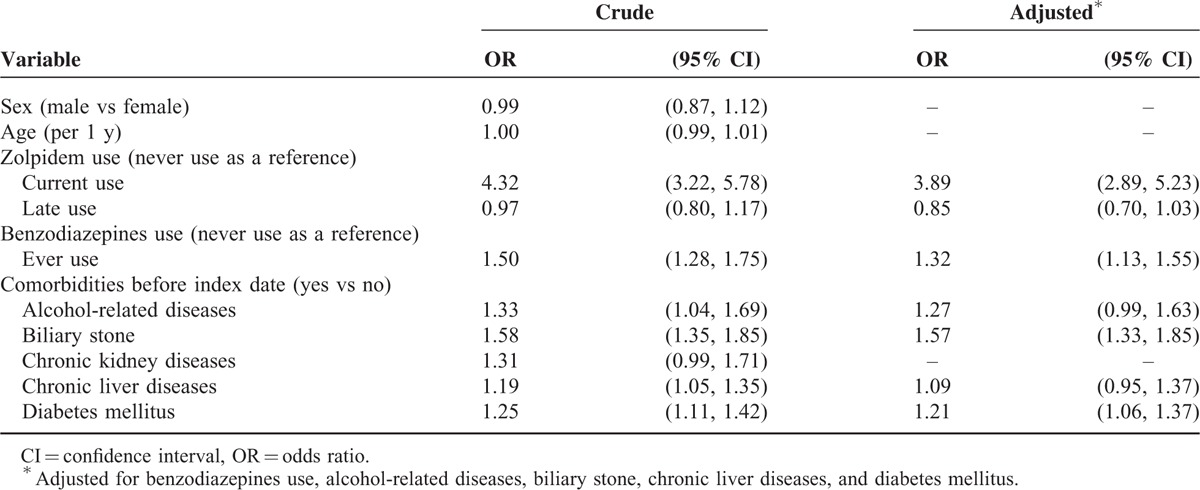
OR and 95% CI of Pyogenic Liver Abscess Associated With Zolpidem Use, Benzodiazepines Use, and Other Comorbidities

### Average Daily Dose of Current Use of Zolpidem and Risk of Pyogenic Liver Abscess

Table 3 shows the association between average daily dose of current use of zolpidem and the risk of pyogenic liver abscess. After adjustment for possible confounding factors, the adjusted OR of pyogenic liver abscess was 2.13 in low dose group with average daily dose ≤10 mg (95% CI 1.46, 3.10, *P* value of test for trend <0.001), when compared with those with never use of zolpidem. The adjusted OR increased to 13.8 in high dose group with average daily dose >10 mg (95% CI 7.60, 25.1, *P* value of test for trend <0.001).

**TABLE 3:**
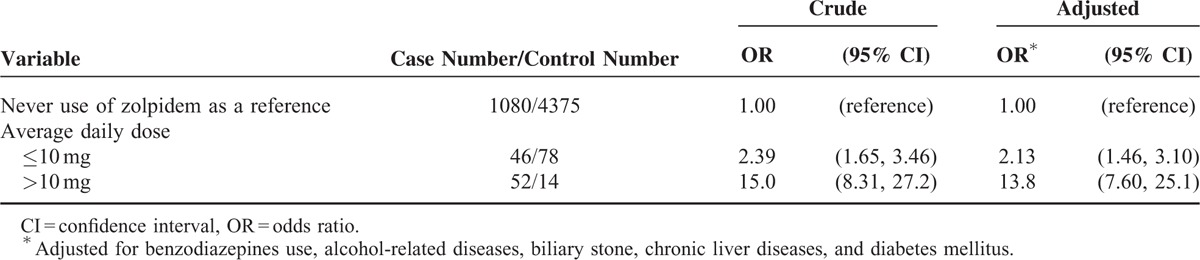
OR and 95% CI of Pyogenic Liver Abscess Associated With Average Daily Dose of Current Use of Zolpidem

## DISCUSSION

This is the first population-based case-control study discloses that people with current use of zolpidem were associated with the increased odds of pyogenic liver abscess (adjusted OR 3.89), but those with late use of zolpidem did not have the increased odds. Given zolpidem only being a short elimination half-life with no active metabolite and no accumulating effect,^3,31^ our findings suggest that only people continuing use of zolpidem would have the risk. Those who had ever used zolpidem but not using it now would not have the risk. In additional analysis, we noted that people with average daily dose of zolpidem >10 mg had higher odds of pyogenic liver abscess than those with average daily dose ≤10 mg (adjusted OR 13.8 vs 2.13). This finding means that there is a dose-dependent effect of zolpidem on risk of pyogenic liver abscess. In addition, we also noted that among people with current use of zolpidem, use of extended-release zolpidem was not associated with the increased odds of pyogenic liver abscess. Only use of nonextended-release zolpidem was associated with the increased odds of pyogenic liver abscess (table not shown).

To date, no prospective clinical trial discloses that zolpidem can induce infections. Only 2 observational studies have disclosed that zolpidem might be associated with some infections,^13,14^ but pyogenic liver abscess was not included. One case report disclosed multiple aseptic cutaneous abscesses on the forearms and feet induced by self-injection of powdered zolpidem in a young female addict.^32^ The U.S. Food and Drug Administration has disclosed that since 2002 to 2012, 13 people (0.03%) had liver abscess among 43,174 people reporting to have side effects when taking zolpidem, but the causal–effect relationship was not confirmed.^33^ Therefore, the potential mechanism of the association between zolpidem use and pyogenic liver abscess cannot be totally determined by this observational study and the available literature. As we know, zolpidem is mainly used to treat patients with insomnia. The literature discloses that insomnia is associated with the decreased immune function,^34,35^ which would further increase the probability of infection. Therefore, the observed association of this study may be confounded by the association between insomnia and pyogenic liver abscess. Because the number of insomnia patients without using any hypnotic agent was too small in this study, it is considerably difficult to explore whether insomnia patients without using any hypnotic agent could have an association with pyogenic liver abscess. Similarly, it is also very difficult to include patients with only zolpidem use but without diagnosis of insomnia for analysis. Owing to no definite evidence disclosing that zolpidem can induce infections, whether insomnia is really associated with pyogenic liver abscess or people with current use of zolpidem have other unfound factors to be associated with pyogenic liver abscess cannot be completely illustrated. It indicates a future research direction on this issue. In addition, benzodiazepines such as diazepam are associated with immune suppression, which may further increase infection risk.^15,16^ We think that zolpidem may have a similar effect on immune suppression like benzodiazepines, which further increase the risk of pyogenic liver abscess.

Some limitations should be discussed in this present study. First, due to the natural limitation of the claim database, we did not know whether people actually took zolpidem of not. The next more rational step was to use prescriptions for analysis. Second, although a differential misclassification of ICD-9 code 572.0 for pyogenic liver abscess may be present due to the natural limitation of the claim database, we included patients only with primary discharge diagnosis of pyogenic liver abscess for analysis. Therefore, the diagnosis accuracy of ICD-9 code 572.0 for pyogenic liver abscess should be reliable. Third, although a cohort study could be performed using this robust database, there might be an immortal time bias. That is why we performed a case-control study instead of a cohort study. Fourth, although the cases and controls were matched with comorbidities, some comorbidities still could not be totally controlled (Table 1). We made an additional analysis to decrease the confounding effects caused by comorbidities. Even in absence of biliary stone and diabetes mellitus, people with current use of zolpidem alone were still associated with the increased odds of pyogenic liver abscess (adjusted OR 6.12, 95% CI 3.65, 10.3). Fifth, no any epidemiological study focuses on this topic. We cannot compare our findings with others. The causal–effect relationship between zolpidem use and pyogenic liver abscess cannot be confirmed in a case-control study. A further prospective research is needed to confirm our findings. Sixth, the literature shows that nonalcoholic fatty liver disease is associated with the increased risk of drug-induced liver injury through mitochondrial dysfunction.^36,37^ No specific ICD-9 code for nonalcoholic fatty liver disease is available. We cannot include nonalcoholic fatty liver disease for analysis. Further research is needed to illustrate whether nonalcoholic fatty liver disease also makes the liver more vulnerable to invasion of infective agents.

Some strengths of this study should be addressed. Because there is a relative paucity of information on this topic, this article provides the updated evidence to physicians. The methodology and analysis seem to be relatively reasonable. The results are of clinical importance.

We conclude that current use of zolpidem is associated with the increased risk of pyogenic liver abscess, with a dose-dependent effect. Physicians should take the risk of pyogenic liver abscess into account when prescribing zolpidem.
